# Upregulation of miR-34a-5p, miR-20a-3p and miR-29a-3p by Onconase in A375 Melanoma Cells Correlates with the Downregulation of Specific Onco-Proteins

**DOI:** 10.3390/ijms23031647

**Published:** 2022-01-31

**Authors:** Elisa De Tomi, Rachele Campagnari, Elisa Orlandi, Alessia Cardile, Valentina Zanrè, Marta Menegazzi, Macarena Gomez-Lira, Giovanni Gotte

**Affiliations:** 1 Department of Neuroscience, Biomedicine and Movement Sciences, Biology and Genetics Section, School of Medicine, University of Verona, I-37134 Verona, Italy; elisa.detomi@univr.it (E.D.T.); elisa.orlandi@univr.it (E.O.); macarena.gomezlira@univr.it (M.G.-L.); 2 Department of Neuroscience, Biomedicine and Movement Sciences, Biochemistry Section, School of Medicine, University of Verona, I-37134 Verona, Italy; rachele.campagnari@univr.it (R.C.); alessia.cardile@studenti.univr.it (A.C.); valentina.zanre@univr.it (V.Z.); giovanni.gotte@univr.it (G.G.)

**Keywords:** ribonuclease, cyclin D1, cyclin A2, HIF1α, PDK1, CREB, SIRT1, SOX2, Fra1, AXL, cMet

## Abstract

Onconase (ONC) is an amphibian secretory ribonuclease displaying cytostatic and cytotoxic activities against many mammalian tumors, including melanoma. ONC principally damages tRNA species, but also other non-coding RNAs, although its precise targets are not known. We investigated the ONC ability to modulate the expression of 16 onco-suppressor microRNAs (miRNAs) in the A375 BRAF-mutated melanoma cell line. RT-PCR and immunoblots were used to measure the expression levels of miRNAs and their regulated proteins, respectively. In silico study was carried out to verify the relations between miRNAs and their mRNA targets. A375 cell transfection with miR-20a-3p and miR-34a-5p mimics or inhibitors was performed. The onco-suppressors miR-20a-3p, miR-29a-3p and miR-34a-5p were highly expressed in 48-h ONC-treated A375 cells. The cytostatic effect of ONC in A375 cells was mechanistically explained by the sharp inhibition of cyclins D1 and A2 expression level, as well as by downregulation of retinoblastoma protein and cyclin-dependent-kinase-2 activities. Remarkably, the expression of kinases ERK1/2 and Akt, as well as of the hypoxia inducible factor-1α, was inhibited by ONC. All these proteins control pro-survival pathways. Finally, many crucial proteins involved in migration, invasion and metastatic potential were downregulated by ONC. Results obtained from transfection of miR-20a-3p and miR-34a-5p inhibitors in the presence of ONC show that these miRNAs may participate in the antitumor effects of ONC in the A375 cell line. In conclusion, we identified many intracellular downregulated proteins involved in melanoma cell proliferation, metabolism and progression. All mRNAs coding these proteins may be targets of miR-20a-3p, miR-29a-3p and/or miR-34a-5p, which are in turn upregulated by ONC. Data suggest that several known ONC anti-proliferative and anti-metastatic activities in A375 melanoma cells might depend on the upregulation of onco-suppressor miRNAs. Notably, miRNAs stability depends on the upstream regulation by long-non-coding-RNAs or circular-RNAs that can, in turn, be damaged by ONC ribonucleolytic activity.

## 1. Introduction

Onconase (ONC) is a 104 AA and 11.8 kDa enzyme, first extracted from the leopard frog (*Rana pipiens*) oocytes or early embryos [1]. Notwithstanding being amphibian, ONC is included in the mammalian pancreatic-type secretory ribonucleases super-family [2] because it exerts ribonucleolytic activity involving a His-Lys-His active site like the other super-family members. Moreover, ONC displays high structural affinity with them and with the proto-type RNase A [3,4]. Notably, ONC exerts both cytostatic and cytotoxic effects in many cancer cell types and inhibits tumor growth in animals [5]. It has been the first RNase tested in Phase II and III clinical trials for non-small cell lung cancer and unresectable malignant mesothelioma, respectively [6,7]. Remarkably, co-administration of ONC with tamoxifen, cisplatin, vincristine or doxorubicin results in an increased toxicity for cancer cells [8,9].

Importantly, ONC displays tumor selectivity accompanied with higher toxicity for malignant than for normal cells. This depends on the presence on the ONC surface of many positive charges, which can better interact with cancer cells that are in turn characterized by more negative charged membranes than normal cells [10]. In addition, the enzyme exhibits high conformational stability [11], very low protease responsiveness [12], and ability to evade the inactivating intracellular ribonuclease inhibitor [13,14]. 

Although the ONC antitumor activity mechanism is not well understood yet, it is indeed strictly dependent on ONC cytosolic internalization and on the catalytic degradation of intracellular RNA [1]. However, ONC displays differential RNA target specificity, leaving ribosomal RNA (rRNAs) and messengers RNA (mRNAs) largely intact [14,15]. Instead, transfer RNA (tRNA) appears to be a substrate susceptible to ONC action [14,15]. This is true, although the protein synthesis inhibition, consequent to tRNA degradation, cannot explain the expression increase of, for example, the cyclin-dependent kinase inhibitors P16, P21 and P27 in U937 lymphoma cells [16], hence indicating that tRNA is not the unique RNA species damaged by ONC. Moreover, in vitro studies reported that the ONC cytotoxic effect becomes appreciable only after a long incubation time, i.e., about 48 h from its administration in the culture medium [17], in which ONC triggers a sequence of a multiplicity of gene and protein repression or induction, finally leading to cell injury. 

Besides the transcription factors able to elicit gene transcription, protein expression regulation depends on the stability of mRNAs that are, in turn, regulated by other RNA species. It is peculiar that the majority of the human genome is transcribed even if only very few of the transcribed genes code for proteins. Remarkably, the large amount of non-coding RNA species (ncRNAs) is now considered to display a key role in health and disease by up- or down-regulating protein expression [18]. The smaller ncRNAs include micro RNAs (miRNAs) and circular RNAs (circRNAs), in addition to the classical RNA species (e.g., rRNAs and tRNAs) [18]. The longer ones are called long-non-coding RNAs (lncRNAs), a heterogenous class of RNAs which are less conserved than protein-coding RNAs, but their expression displays high tissue specificity [18]. Both lncRNAs and circRNAs could sponge and affect miRNAs that, in turn, impact mRNA stability and translation, and consequently, protein expression [19]. Thus, by targeting miRNAs, or the other ncRNAs, ONC can regulate the expression of intracellular proteins [20]. Accordingly, ONC can affect the expression of miRNAs through which protein expression is modulated in mesothelioma cell lines [21,22].

MiRNAs can display either tumor-suppressing or tumor-promoting effects, depending on the function of target genes involved in cancer [19]. Cumulative evidences showed a heavily altered miRNA expression in human cancer, in which onco-suppressor miRNAs can be downregulated, while tumor-promoting miRNAs can be highly expressed [23]. Additionally, both lncRNA and circRNA species are differently expressed in tumors and could become targets for anticancer therapy [18,19,23]. 

Melanoma is an aggressive cancer associated with the poorest prognosis. Activating mutations in the BRAF gene are the most frequent genomic alterations, present in more than half of all melanoma cases [24]. Unfortunately, target therapy with BRAF-inhibitors or MEK-inhibitors does not exclude recurrence of the malignancy and the incoming of therapy resistance [24]. Recently, we reported a significant anti-melanoma activity exerted by low micromolar ONC concentrations in both A375 and MeWo human malignant melanoma cell lines [12,17,25]. In addition, ONC enhanced its antitumor efficacy in cells pre-treated with other drugs. Actually, in a A375 subpopulation of cells characterized with acquired resistance to the BRAF-inhibitor dabrafenib, we showed that ONC incubation increases apoptosis, while it decreases colony formation in soft agar, and cell-invasion capability at a higher extent with respect to parental A375 cells [17]. We also registered the ONC ability to hinder expression and activity of nuclear factor-κappa-B (NF-κB) [17,25], as well as of signal transducer and activator of transcription (STAT)-3 [12]. In the present work, we investigate how other intracellular targets are affected by ONC in the A375 cell line that is characterized by the BRAF^V600E^ mutation. Besides, we principally aim to determine if the ONC anti-melanoma effect is due to the induction of some onco-suppressive miRNA species that can be in turn associated with the expression of proteins encoded by their target genes. 

Therefore, we initially tested the expression level of several miRNAs in the A375 cell line after ONC incubation, observing the induction of three miRNAs generally considered to act as tumor suppressors. The results obtained were confirmed in FO1 cells, another BRAF-mutated melanoma cell line. 

MiR-34a-5p (ENSG00000284357) is a tumor suppressor miRNA characterized in a variety of tumors, where it plays a pivotal role in regulating cancer-related processes, such as cell proliferation, apoptosis, epithelial-mesenchymal transition (EMT), and metastasis [26,27,28,29,30].

MiR-20a (ENSG00000283762) has been extensively studied in different malignancies [31,32,33,34], but so far, miR-20a-3p, the passenger strand of the mature miRNA, has been rarely reported. Three different studies registered an altered expression of this miRNA in patients affected by β-thalassemia, breast cancer, and psoriasis [35,36,37], in which a decreased expression of miR-20a-3p emerged. Besides, both miR-20a-5p and miR-20a-3p are under-represented in the murine B16 melanoma cell line in comparison with non-malignant HaCaT cells, and the restoration of miR-20a expression in malignant B16 cells attenuated their growth [38].

Another miRNA generally considered to be a tumor suppressor is miR-29a-3p (ENSG00000284032); it belongs to the miR-29 family that controls many oncogenic genes/pathways in several cancers [39,40,41,42]. Moreover*,* miR-29a has been shown to be downregulated in five melanoma cell lines, whereas its over-expression suppressed A375 cell growth, migration and invasion by blocking Wnt/β-catenin and NF-κB pathways [43].

To deepen the comprehension of the regulatory mechanism of ONC, we constructed a kind of miRNA–mRNA network. An R package called multiMiR [44] was created to verify the predicted and validated relations existing between the significantly inducted miRNAs and their mRNA targets related to cell cycle regulation, metabolism and signaling pathways activated in malignancy.

Finally, considering that miR-20a-3p and miR-34a-5p were the highest expressed, we investigated their functional role in ONC-treated A375 cells by transfecting cells with miR-20a-3p and miR-34a-5p inhibitors. The results obtained globally indicate that the miRNAs studied here play an important role in A375 cells treated with ONC.

## 2. Results

### 2.1. ONC Downregulates the Expression Level of Key Proteins Involved in A375 Cell Cycle Progression

We previously reported that ONC exerts a strong cytostatic effect in the A375 BRAF-mutated cell line by reducing the Br-deoxy-uridine incorporation into DNA in a time- and concentration-dependent manner [17]. Actually, A375 cell proliferation was reduced by about 50–60% after 48 and 72 h incubation with 1 µM ONC [17]. In this work, we investigate the mechanism of this cytostatic effect in the same melanoma cell line.

The hyperphosphorylation of the retinoblastoma protein (pRB) is required for the transition of the cell cycle from G1- to S-phase [45], and the cell cycle progression is further regulated by a sequential and coordinated rise and fall of cyclin-dependent kinases (Cdks) activity [46].

**Figure 1:**
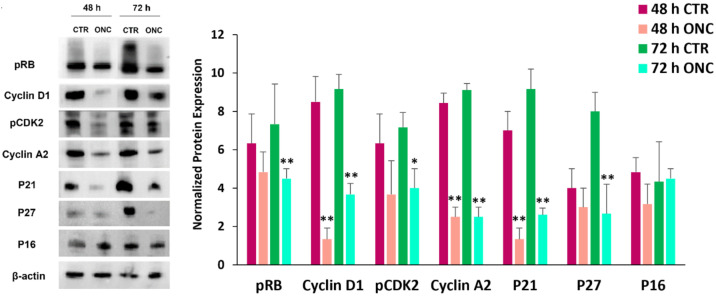
Immunoblot data attesting the onconase (ONC) inhibitory effects on cell cycle-related protein expression. A375 melanoma cells were cultured in the presence or absence of 1 µM ONC for 48 or 72 h. Left: immunoblots showing the expression levels of cell cycle-related proteins; right: histograms reporting the mean values ± S.D. of protein expression level measured by densitometry and deriving from three to four independent experiments. All comparisons were performed vs. each control sample after normalization with β-actin expression; * *p* < 0.05, ** *p* < 0.01. pRB, phosphorylated form of retinoblastoma protein; pCDK2, phosphorylated form of cyclin-dependent kinase-2; P21/Cip1; p27/Kip1; p16/Ink4A.

Immunoblot data showed here a robust reduction of both activated forms of RB (pRB) and Cdk2 (pCdk2) in A375 cells treated with 1 µM ONC for 48 or 72 h (Figure 1). Because RB hyperphosphorylation is principally triggered by cyclin D1, and cyclin A2/Cdk2 binding is required for cells to enter S-phase, we investigated both cyclin D1 and cyclin A2 expression level, registering a powerful reduction of both after 48 h from ONC administration, and this reduction was almost totally maintained for a further 24 h (Figure 1). 

Altogether, the immunoblot data suggest that ONC can hinder A375 cell cycle progression, firstly by inhibiting cyclin D1 expression, which controls RB hyperphosphorylation and the G1/S transition point. Indeed, the reduction of both Cdk2 phosphorylation and cyclin A2 expression can further hamper cell cycle progression. 

P21/Cip1, P27/Kip1 and P16/Ink4A are inhibitors of Cdks [47] and their increase could be responsible for the pRB and pCdk2 low expression levels. Conversely, immunoblot showed that the expression of P16 did not change at all, while the protein amount of both P21 and P27 was sharply lowered (Figure 1). In brief, the ONC-elicited blockage of cell cycle progression cannot result from a P21, P27 or P16 different expression.

### 2.2. ONC Differently Affects the Expression Level of Proteins Involved in A375 Cell Survival Signaling and Metabolism

Activation of the mitogen-activated protein kinase/extracellular signal-regulated kinase (MAPK/ERK) pathway in BRAF-mutated melanoma cells, as A375 cells, can promote hypoxia inducible factor-1 alpha (HIF1α) expression, hence leading to a high glycolytic rate [48]. HIF1α, as well as phosphoinositide 3-kinase/protein kinase B/mammalian target of rapamycin (PI3K/Akt/mTOR) pathways are crucial regulators of cancer cell proliferation and glycolytic metabolism [49]. Furthermore, pyruvate dehydrogenase kinase 1 (PDK-1) is able, upon its inhibitory phosphorylating effect on pyruvate dehydrogenase (PDH), to trigger the switch to glycolysis by hindering the oxidative metabolism of pyruvate [48]. 

**Figure 2:**
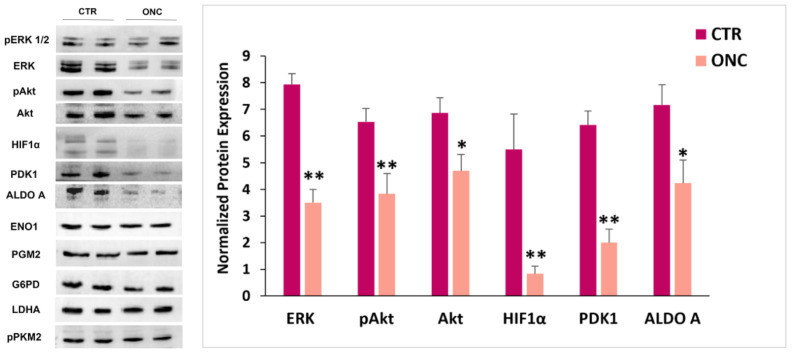
ONC effects on the expression of proteins involved in cell proliferation signaling and metabolism. A375 melanoma cells were cultured for 48 h in the presence or absence of 1 µM ONC. Left: immunoblots showing the expression levels of extracellular signal-regulated kinase (ERK) and protein kinase B (Akt) and their active forms, and of many enzymes involved in metabolism; right: histograms reporting the mean values ± S.D. of the protein expression level measured by densitometry and deriving from three to four independent experiments. All comparisons were performed vs. each control sample after normalization with enolase-1 (ENO1) and phospho-glucomutase-2 (PGM2) expression; * *p* < 0.05, ** *p* < 0.01. HIF1α, hypoxia inducible factor 1 alpha; PDK1, pyruvate dehydrogenase kinase 1; ALDO A, aldolase A; G6PD, glucose-6-phosphate dehydrogenase; LDHA, lactate dehydrogenase A; pPLM2, phosphorylated form of pyruvate kinase M-2.

Here, both ERK1/2 and Akt total proteins expression levels significantly decreased after 48-h incubation with 1 µM ONC. However, the amount of the phosphorylated and active form of ERK1/2 did not change with the treatment, whereas pAkt expression level was decreased (Figure 2). HIF-1α was expressed in A375 cells after 48-h culture at normal oxygen pressure. Remarkably, cell incubation with 1 µM ONC decreased almost totally the HIF1α protein level (Figure 2). Thus, we measured the expression level of some key enzymes to explore if the metabolic phenotype of the A375 cell line was altered.

The expression level of glucose-6-phosphate dehydrogenase (G6PD), phospho-glucomutase-2 (PGM2), enolase-1 (ENO1), lactate dehydrogenase A (LDHA), and the phosphorylated form of pyruvate kinase M-2 (pPKM2) did not vary upon 48-h ONC incubation, so that these enzymes were further exploited as housekeeping proteins for normalization. Conversely, either aldolase A (ALDO A) or PDK1 significantly decreased their expression level (Figure 2).

### 2.3. ONC Treatment Downregulates the Expression of Key Proteins Involved in A375 Melanoma Cells Metastatic Potential

We recently reported that ONC is able to affect A375 cells colony formation in soft agar, as well as metalloproteinase (MMP)-2 activity and wound closure time-course after scratching [17]. 

Zonula occludens protein-1 (ZO1) is involved in the regulation of cell–cell contacts [50]. Sirtuin 1 (SIRT1) induces EMT and facilitates melanoma metastasis [51,52]. SRY (sex determining region Y)-box 2 (SOX2) is an embryonic stem cell transcription factor that is associated with dermal invasion of melanoma cells [53]. Urokinase plasminogen activator receptor (uPAR) has been associated with invasion and metastasis in melanoma [54,55], while the cAMP response element-binding protein (CREB) is a transcription factor playing an important role in the acquisition of the metastatic phenotype of human melanoma cells [56].

**Figure 3:**
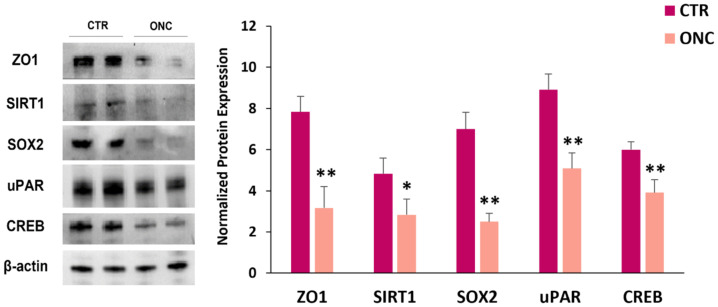
Immunoblot data attesting the ONC inhibitory effects on the expression of proteins involved in cell migration, invasion and tumor progression. A375 melanoma cells were cultured for 48 h in the presence or absence of 1 µM ONC. Left: immunoblots showing the expression levels proteins involved in cell invasive potential; right: histograms reporting the mean values ± S.D. of the protein expression level measured by densitometry and deriving from three to four independent experiments. All comparisons were performed vs. each control sample after normalization with β-actin expression; * *p* < 0.05, ** *p* < 0.01. ZO1, zonula occludens protein-1; SIRT1, sirtuin 1; SOX2, SRY (sex determining region Y)-box 2; uPAR, urokinase plasminogen activator receptor; CREB, cAMP response element-binding protein.

Figure 3 shows a significant decrease of ZO1, SIRT1, SOX2, uPAR and CREB expression levels in A375 cells treated with ONC for 48 h. 

### 2.4. MiRNAs Are Modulated by ONC in A375 and FO1 Melanoma Cells

**Figure 4:**
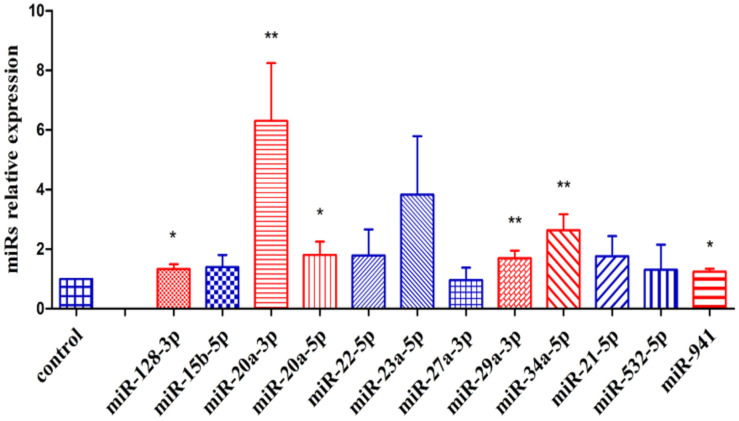
Relative expression of miRNAs after 48 h incubation of A375 cells with ONC. Cells were cultured for 48 h after 1 µM ONC administration in the culture medium. Red color bars refer to the onco-suppressor miRNAs that were upregulated by ONC at a statistically significant level; the blue ones refer instead to miRNAs whose expression level was not significantly different from the one relative to the untreated control. The mean values ± S.D. of miRNAs expression level measured by RT-PCR and deriving from three independent experiments are shown. All comparisons were performed vs. each control sample after normalization to miR-191 expression; * *p* < 0.05, ** *p* < 0.01.

Initially, the effect of ONC in A375 cells was evaluated on the expression of 24 miRNAs associated with different types of cancer, as are onco-miRNAs or tumor suppressive miRNAs (Table S1, see Supplementary Materials). From these results, we selected for further analysis the two down-regulated miRNAs and the up-regulated miRNAs that showed an at least 2.5-fold change expression. Then, two other independent experiments were performed, and Figure 4 shows that the miRNAs displaying the highest significant upregulation (*p* < 0.01) were miR-20a-3p, miR-29a-3p, and miR-34a-5p. The other three miRNAs, miR-128-3p, miR-20a-5p and miR-941, were upregulated, but with a lower *p* value (Figure 4). Conversely, a first analysis showed that two miRNAs were downregulated, but this result was not confirmed either in a second or a third experiment.

Additional experiments were performed also in the FO1 melanoma cell line [57]. Firstly, we tested if FO1 cells were sensitive to ONC antitumor effect. A sulforhodamine B viability assay was carried out after incubating FO1 cells for 72 h in the presence of different concentrations of ONC. The results, reported in Figure S1 (Supplementary Materials), show a concentration-dependent FO1 cell growth inhibition. Next, miR-20a-3p, miR-20-5p, miR-34a-5p, mirR-128-3p, and miR-29a-3p expression levels were measured in FO1 cells after 48 h incubation with 1 µM ONC, compared with not-treated cells. Data reported in Figure S2 (Supplementary Materials) show a significant over-expression of miR-20a-3p, miR-34a-5p and miR-29a-3p, in agreement with the expression level of the same miRNAs in A375 cells. 

### 2.5. Predicted microRNA-Target Interactions 

**Figure 5:**
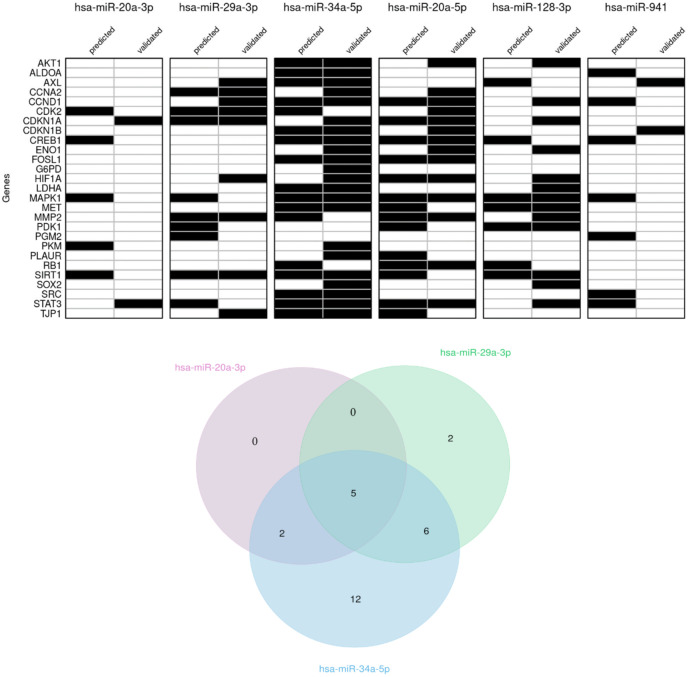
miRNA-target interaction. Top: table shows predicted and validated miRNA-target interactions. Bottom: Venn diagram for miR-20a-3p, miR-29a-3p, and miR-34a-5p. Genes targeted by all three miRs: CDK2, CDKN1A, MAPK1, SIRT1, STAT3.

We next queried different databases to determine the interactions occurring between upregulated miRNAs and mRNAs of proteins involved in cell cycle regulation, metabolism, and signaling pathways activated in melanoma. In Figure 5, we reported the genes that in literature have been predicted and/or validated as a targets [58] of each miRNA that in our study resulted to be upregulated.

### 2.6. ONC-Elicited Downregulation of cMet and AXL Tyrosine-Kinase Receptors, and of Fra1 Transcription Factor Correlates in A375 Cells with the Upregulation of miR-34a-5p and miR-20a-3p Expression

**Figure 6:**
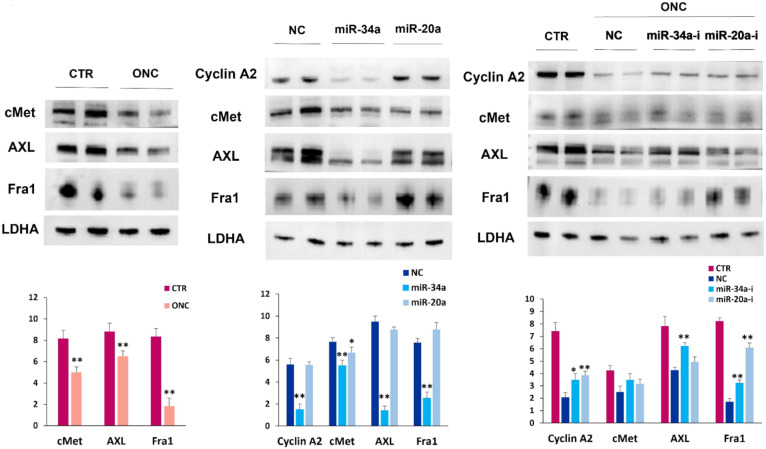
Immunoblots and densitometric data representing the correlation between mesenchymal–epithelial transition factor (cMet), tyrosine-protein kinase receptor UFO (AXL), Fos-related antigen 1 (Fra1) and cyclin A2 protein level and miR-34a-5p and miR-20a-3p. Left panel: A375 melanoma cells were treated for 48 h with or without ONC; expression of cMet, AXL, and Fra1 proteins. Central panel: effects of the overexpression (72 h) of miR-34a-5p and miR-20a-3p on cMet, AXL, Fra1 and cyclin A2 protein level in untreated cells. Right panel: effects of miR-34a-5p and miR-20a-3p inhibitors on cMet, AXL, Fra1 and cyclin A2 protein levels on ONC-treated A375 cells transfected with 50 nM miRNAs inhibitors for 72 h. In the upper part of each panel, immunoblots show the expression levels of the proteins. In the lower part, histograms report the relative mean values ± S.D. of protein expression level measured and deriving from three independent experiments. All comparisons were performed vs. each control sample after normalization with LDHA expression; * *p* < 0.05, ** *p* < 0.01.

We analyzed the expression of the mesenchymal–epithelial transition factor (cMet), a cell surface tyrosine-kinase receptor constitutively active in melanoma cells [59,60]. We also investigated the expression of the tyrosine-protein kinase receptor UFO (AXL), an oncoprotein involved in metastasis and resistance to various anti-cancer drugs [61], as well as the expression of Fos-related antigen 1 (Fra1) protein belonging to the transcription factor activator protein 1 (AP1) complex [62]. Fra1 expression correlates with cell transformation to a more invasive phenotype [63]. The expression level of AXL and c-Met tyrosine-kinase receptors, as well as of Fra1 transcription factors, was lower in ONC-treated A375 cells (Figure 6, left panel).

The miR-34a-5p and miR-20a-3p, the highest expressed miRNAs upon ONC treatment, were chosen to verify if cMet, AXL, Fra1 and also cyclin A2 were affected by these miRNAs in A375 cells. Thus, we transfected A375 cells with miR-34a-5p, miR-20a-3p or negative control mimics, and protein expression level was measured by immunoblot after 72 h. As shown in the central panel of Figure 6, overexpression of miR-34a-5p was able to decrease the expression level of all the proteins analyzed. MiR-20a-3p overexpression partially downregulated cMet expression but showed no effect on AXL, Fra1 and cyclin A2 (Figure 6, central panel). 

To further verify if the downregulation of cMet, AXL, Fra1 and cyclin A2 protein expression elicited by ONC is related to the upregulation of these miRNA species, ONC-treated A375 cells were transfected with miR-34a-5p or miR-20a-3p inhibitors, or with negative control. Seventy-two hours after the miR-inhibitor transfection and ONC treatment, immunoblot results showed that the miR-34a-5p inhibitor could partially revert the effect of ONC on cyclin A2, AXL, cMet and Fra1, indicating the participation of miR-34a-5p in the expression decreases elicited by ONC (Figure 6, right panel). MiR-20a-3p inhibitor partly reverts the ONC effect on cyclin A2 and Fra1 expression level (Figure 6, right panel), indicating that miR-20a-3p may take part in the regulation of the last-mentioned proteins.

## 3. Discussion

We presently investigate the molecular mechanism underlying the high cytostatic effect of ONC in A375 melanoma cells that we had previously described [17]. Our data suggest that ONC can hinder cell cycle progression by inhibiting RB hyperphosphorylation and Cdk2 activity, as well as by reducing the expression level of D1 and A2 cyclins, which are involved in G1/S and S/G2 cell cycle phases (Figure 1).

Since P21/Cip1, P27/Kip1 and P16/Ink4A are Cdks inhibitors [47], we expected that ONC could lead to the upregulation of their protein expression, as reported by Juan et al. [16] for the U937 lymphoma cell line. Conversely, the lowered expression level of P21 and P27 found in A375 cells treated with ONC (Figure 1) cannot support this hypothesis.

ONC is an amphibian RNase characterized by a sharp anticancer activity related with its ability to digest intracellular RNA species. tRNAs are ONC targets [14,15], but ncRNA species are targets as well [20,21,22], the latter ones being able to regulate gene expression. Hence, we assumed that ONC can decrease cell cycle-related protein expression by upregulating some miRNAs. Among the onco-suppressor miRNAs whose expression was measured in A375 cells, miR-20a-3p, miR-29a-3p and miR-34a-5p were the most significantly upregulated after 48 h ONC treatment (Figure 4). The same miRNAs were also overexpressed in FO1 melanoma cells (Figure S2).

Actually, cyclin D1 and cyclin A2 may be targets of miR-29a-3p and miR-34a-5p, whereas CDK2 may be a target of miR-20a-3p, miR-29a-3p and miR-34a-5p (Figure 5). The inhibition of their expression can justify the hindering of cell cycle progression [17]. The downregulation of P21 and P27, although not explaining the cell cycle hindering, is not surprising considering that P21 may be a target of all three overexpressed miRNAs, and P27 may be a target of miR-34a-5p (Figure 5). 

Many studies have evaluated the miRNAs capability to affect cellular growth. For example, Shao et al. [64] reported that miR-29a-3p downregulation promotes glioma cell proliferation. Again, it has been demonstrated that an ectopic expression of miR-34a induces cell cycle arrest [65]. Moreover, transfection of miR-34a into uveal melanoma cells led to a significant decrease of cell growth and migration [66]. A recent study reported that A375 cell proliferation was decreased after miR-34a overexpression, and increased when miR-34a expression was suppressed [67]. In addition, miR-34a also inhibits melanoma tumor growth in a mouse melanoma model [67].

We now registered that the A375 cells transfection with miR-34a-5p or miR-20a-3p inhibitors in the presence of ONC partially reverts the ONC-elicited decrease in cyclin A2 expression (Figure 6). This suggests that the ONC cytostatic effect could be mediated by the overexpression of these tumor suppressive miRNAs. 

Kuphal et al. registered a marked HIF1α activity in melanoma cell lines under normoxic conditions [68]. Accordingly, ^18^fluorine-deoxyglucose positron-emission tomography (^18^FDG-PET) data show that the glycolytic phenotype is increased in BRAF-mutated melanoma patients [69]. 

Our immunoblot data report that ONC administration almost suppresses the expression of HIF1α transcription factor present in A375 control cells, even if they were cultured at normal oxygen pressure. Recent studies revealed that HIF1α can be induced by different signaling pathways, such as PI3K/Akt/mTOR, RAS/RAF/MEK/ERK, JAK/STAT, and NF-κB pathways controlling melanoma tumor growth, metabolism, motility, and apoptosis evasion [70]. Previously, we showed that ONC decreases expression and activity of both NF-κB [17,25] and STAT3 [12] in A375 melanoma cells. In the present study, we registered the ONC ability to lower Akt expression and phosphorylation levels, as well as the total protein expression of the kinases ERK1/2 (Figure 2). Thus, the ONC-elicited downregulation of these signaling pathways may altogether contribute to suppress HIF1α protein expression. 

The ONC-triggered downregulation of HIF1α led us to investigate other proteins which might impact the metabolic status of A375 cells. The expression of many cytosolic enzymes belonging to glucose metabolism was not affected by ONC, excluding ALDO A that is less expressed and may be a target of miR-34a-5p. 

It is known that the mitochondrial enzyme PDH is a guardian enzymatic complex that connects glycolysis with oxidative metabolism [48]. The PDH activity is inhibited if the enzyme is phosphorylated by its regulatory kinases PDKs and, in cancer cells, PDK1 is transcriptionally induced by HIF1α [71]. Our results show a lower expression level of PDK1, in agreement with the HIF1α decrease, suggesting that the higher activity of PDH could result in increasing the pyruvate oxidative metabolism. Notably, both HIF1α and PDK1 may be targets of miR-29a-3p. HIF1α, whose protein expression is almost suppressed, may also be a target of miR-34a-5p. 

Interestingly, miR-20a-3p showed to be the most widely expressed miRNA upon ONC treatment. In relation to cancer, its decrease has been associated with both breast cancer [36] and pancreatic ductal adenocarcinoma progression [72]. MiR-20a-3p can target different genes, including MAPK1/ERK, STAT3 and CREB1 (Figure 5). Therefore, together with miR-34a-5p, miR-20a-3p can participate in the ONC-elicited growth inhibition, and it can counteract the cancer metabolic phenotype as well. In addition, in silico analysis of the predicted targets (Figure 5) demonstrated that PDK1, HIF1α, and MAPK1 can be targets of miR-29a-3p, suggesting a possible role of miR-29a-3p in metabolism, in agreement with its role in insulin receptor signaling in the liver of diabetic rats [73].

Many oncoproteins are involved in melanoma cell capability to grow in an anchorage-independent manner, as well as to increase motility and to digest extracellular matrix [74]. The higher expression of tumor suppressor miRNAs we found in A375 cells treated with ONC could be a rationale to explain the inhibition of A375 metastatic potential previously shown [17]. Therefore, miR-29a-3p can play a pivotal role, since the inhibition of miR-29a-3p in melanoma cells enhances the colony formation ability. Conversely, its transfection reduces A375 cell growth [43].

The trans-differentiation mechanisms, allowing reversible switches between epithelial and mesenchymal phenotypes by reactivating embryonic transcriptional programs, are major determinants of the cancer cell stem (CSC) phenotype [75,76,77]. 

MiR-34a downregulates many CSC-related transcription factors, including SOX2 in head and neck squamous carcinoma cells [58]. In this previous study, the restoration of miR-34a significantly inhibited EMT and the CSC phenotype, and functionally reduced both the clonogenic and invasive capability [58]. MiR-34a was also found to be involved in the regulation of osteosarcoma dedifferentiation by acting via SOX2 downregulation [78]. SOX2, whose expression is decreased by ONC (Figure 3), is also involved in the resistance to BRAF inhibitors therapy in a proto-oncogene tyrosine-protein kinase Src (Src)- and STAT3-dependent manner [79]. We previously reported a downregulation of STAT3 together with its upstream kinase Src in ONC-treated A375 cells [12]. The restoration of miR-34a in triple-negative breast cancer cell lines inhibited proliferation and invasion by targeting the proto-oncogene Src [80]. 

Histone deacetylase SIRT1 is downregulated by ONC (Figure 3), and it was reported to induce EMT and facilitate melanoma metastasis [51]. SIRT1 is a target of miR-34a, as it has been demonstrated in a mouse melanoma cell line [52]. In cervical cancer, it was reported that miR-29a-3p also targets SIRT1 [81]. 

Importantly, miR-34a inhibited cell migration and invasion by also silencing MMP2 expression, interrupting MMP2-mediated cell motility [82]. These results are in line with our previous data demonstrating the ONC capability to decrease MMP2 activity and motility of A375 cells [17]. 

Tumor progression is also characterized by an increased expression of the uPA/uPAR system. Inhibition of uPAR expression with a specific uPAR antisense oligonucleotide inhibits cell invasion, angiogenesis, metastasis and MMPs [54]. uPAR is downregulated by ONC (Figure 3) and may be a target of miR-34a-5p. 

A key role of CREB in tumor growth and metastasis of human melanoma was demonstrated by using a dominant negative CREB gene mutated within its DNA-binding domain [56]. CREB-dominant-negative-transfected cells displayed a remarkable decrease in their ability to form colonies in agar, suggesting that CREB may be involved in tumorigenicity and metastatic potential of human melanoma cells [56]. Our previous results on ONC ability to decrease colonies in soft agar [17] could also derive from CREB expression decrease (Figure 3), possibly via miR-20a-3p and miR-34a-5p activity (Figure 5). 

Finally, we discuss about the role of cMet, AXL and Fra1, a cluster of proteins highly expressed in melanoma and involved in signaling. Both cMet and AXL belong to the class of cell surface receptors with tyrosine-kinase activity (RTKs). RTKs can amplify the signal from growth factors and transduce it to intracellular kinases, i.e., Src, ERKs, Akt, or to transcription factors such as STAT3, NF-kB, and AP1, leading to a cellular program of invasive growth [59,83]. Indeed, many RTKs, including cMet and AXL, are overexpressed in malignancy, displaying oncogenic promotion of cancer progression and metastatic disease, so that high cMet expression in melanoma samples has been correlated with a poor clinical outcome [59]. In addition, the increase of cMet or AXL signaling can develop drug-resistance, in particular to BRAF or MEK inhibitors [84]. Consequently, a combined BRAF/MEK inhibitor therapy plus a cMet or AXL inhibitor has been considered for enrollment in a clinical trial [85]. Both cMet and AXL may be targets of miR-34a-5p, miR-128-3p and miR-20a-5p (Figure 5).

Downstream of RTKs signaling, Fra1 can be activated. Its transcriptional activity, together with other proteins of the AP1 complex, is involved in most of the steps of metastatic dissemination, including tissue invasion and colonization at distant sites [77]. A recent study showed that Fra-1 overexpression in melanocytes is sufficient to drive pro-tumorigenic features and dedifferentiation [77]. Indeed, silencing FOSL1, i.e., the gene encoding Fra1, decreases pERK1/2 expression, as well as the proliferation rate in A375 and A2058 melanoma cells. This also induces a significant decrease of soft-agar colony formation [63]. The cancer-associated downregulation of multiple oncosuppressor miRNAs contributes to Fra1 accumulation in tumors [77]. Recent studies report the FOSL1 transcript to be a target of miR-34a in many cell lines [63,86,87].

In the left panel of Figure 6, we report the ONC-elicited expression decrease of cMet, AXL and Fra1 in A375 cells. Particularly, we observe a very strong decrease in Fra1 expression level after 48 h from ONC administration. Furthermore, we show a reduced expression of cMet, AXL and Fra1 after transfection of miR-34a-5p mimic, as well as a reduction of cMet expression level overexpressing miR-20a-3p. This indicates that cMet, AXL and Fra1 transcripts may be targets of miR-34a-5p, and cMet may also be a target of miR-20a-3p in the A375 cell line. 

Remarkably, our results, showing that the ONC effect is partially reverted by transfecting miR-34a-5p inhibitor (Figure 6, right panel), suggest that the ONC suppressive effect on the cMet, AXL and Fra1 oncoprotein expression could be mediated by miR-34a-5p. As for Fra1, its expression is reverted by transfecting the miR-20a-3p inhibitor as well (Figure 6, right panel). These data could explain the very powerful effect of ONC in lowering Fra1 protein level.

In summary, our data can help to explain the molecular mechanism underlying the ONC suppressive effect exerted on some important oncoproteins in A375 melanoma cells.

## 4. Materials and Methods

### 4.1. Cell Culture

Melanoma A375 cells (CRL-1619; ATCC, Manassas, VA, USA) were grown in Roswell Park Memorial Institute 1640 (RPMI). FO1 (CRL-12177; ATCC, Manassas, VA, USA) (https://web.expasy.org/cellosaurus/CVCL_GZ38 (accessed on 22 January 2022)). All media were supplemented with 10% heat-inactivated fetal bovine serum, 1% L-glutamine (200 nM solution) and 2% Penicillin-Streptomycin (5000 I.U./mL and 5000 µg/mL solution, respectively). All cell cultures were maintained at 37 °C in a humidified incubator in an atmosphere of air and 5% CO_2_.

### 4.2. ONC Expression and Purification

The plasmid encoding for recombinant ONC (pcDNA-ONC) was used for ONC expression in *E. coli*. The protein was extracted and refolded from inclusion bodies following the protocol described by Notomista et al. [88]. 

Met(-1) was removed by incubating with *P. aeruginosa aminopeptidase* (AAP), at an AAP:ONC = 1:1000 molar ratio. The reaction was protracted for 96 h at 37 °C with ONC dissolved at 0.3 mg/mL in 0.2 M potassium phosphate, pH 8.0, containing 20 µM ZnSO_4_, and then was blocked with 0.01 M EDTA, final concentration. 

The final chromatographic purification was performed after the removal of the Met(-1) residue with a Superdex 75 HR 10/300 SEC column attached to an ÄKTA-FPLC system (GE-Healthcare, Little Chalfont, UK). The accuracy of ONC expression was assessed by measuring the RNase “Kunitz” activity on yeast RNA [89,90]. The Met(-1) removal was detected with mass spectrometry (MS) into a ground steel MALDI target plate of a Bruker Ultraflextreme MALDI-TOF/TOF instrument (Bruker Daltonics, Billerica, MA, USA), of the “Centro Piattaforme Tecnologiche” (CPT), of the University of Verona, Italy.

### 4.3. Cell Incubation with ONC

Thirty thousand cells were seeded in a 24-well plate. After 24 h, ONC was added to a final concentration of 1 µM and incubated for 48 h. Cells were then treated with Trizol reagent (Thermo-Fisher Scientific, Milan, Italy) for total RNA extraction.

### 4.4. RNA Extraction and Reverse Transcription

Total RNA was extracted with Trizol Reagent (Thermo-Fisher Scientific), following the manufacturer’s protocol. For each sample of total RNA, 1 µL was directly reverse-transcribed with the TaqMan Advanced miRNA cDNA Synthesis Kit (Thermo-Fisher Scientific) following the manufacturer’s protocol.

### 4.5. Real-Time PCR

Following reverse-transcription, miRNA expression levels were determined by real-time polymerase chain reaction (RT-PCR). TaqMan Fast Advanced Master Mix and the specific probes for 16 selected miRNAs (Thermo-Fisher Scientific) were used. Normalization of the expression was performed by miR-191-5p. The real-time PCR was performed in the Bio-Rad CFX Connect Real-Time System using the TaqMan Advanced miRNA probes (Thermo-Fisher Scientific, Milan, Italy). 

Relative quantification was calculated by Pfaffl’s formula [91]. Each measurement was carried out in triplicate in three different experiments. Differences in the relative expression levels were analyzed with the GraphPad Prism statistical program, using the non-parametric Mann–Whitney test.

### 4.6. Total Protein Extracts and Sample Preparation for Immunoblot Analysis

A375 cells were seeded in 6 cm Petri dishes (190,300 cell/dish). After 48- or 72-hour ONC incubation, cells were scraped using warm 1× sample buffer (2% SDS, 10% glycerol, 50 mM Tris-HCl, 1.75% β-mercaptoethanol, and bromophenol blue) and boiled at 99 °C for 10 min. Total protein extracts were kept at −80 °C until use.

### 4.7. Immunoblot Analysis

Protein extracts were analyzed with a 7.5 or 10% polyacrylamide SDS-PAGE and transferred to a polyvinylidene difluoride membrane (PVDF, Thermo Fisher Scientific, Waltham, MA, USA). Membranes were blocked at RT for 1 h with TBST buffer (10 mM Tris-HCl pH 7.5, 100 mM NaCl, 0.1% Tween20) containing 5% bovine serum albumin (BSA, Serva Electrophoresis GmbH, Heidelberg, Germany). Then, they were incubated on a shaker, overnight (ON) at 4 °C, with 5% BSA solution containing primary antibody against pRb (#8516, 1:2000), pThr202/Tyr204ERK (#9101, 1:1000), LDHA (#3582, 1:1000), pSer473Akt (#4060, 1:2000) (Cell Signaling Technology, Danvers, CO, USA); Akt (GTX121937, 1:3000), ALDO A (GTX101408, 1:3000), Cyclin D1 (GTX106624,1:3000), Cyclin A2 (GTX-103042, 1:3000), cMet (GTX100637), CREB (GTX112846, 1:3000), pThr160Cdk2 (GTX-133862, 1:3000), p21 (GTX-629543, 1:3000), p27 (GTX100446, 1:3000), ENO-1 (GTX101803, 1:3000), ERK1/2 (GTX134462, 1:3000), G6PD (GTX101218, 1:3000), HIF1α (GTX127309, 1:3000), PDK1 (GTX105999, 1:3000), PGM2 (GTX119168, 1:3000), pSer37PKM2 (GTX133886, 1:3000), SOX2 (GTX101507, 1:3000), Src (GTX134412, 1:3000), uPAR (GTX100467, 1:3000), ZO-1 (GTX108592, 1:3000) (Genetex, Alton Pkwj Irvine, CA, USA); AXL (813196-1-AP) (Proteintech, Manchester, UK); and SIRT1 (Sc-74465, 1:1000) (Santa Cruz Biotechnology, Dallas, TX, USA). Membranes were washed three times with TBST buffer for 30 min, and then incubated for 1 h with a horseradish peroxidase-conjugated secondary antibody (anti-rabbit 1:6000, Cell Signaling Technology). They were then washed another three times for 30 min with TBST buffer. Protein extracts were normalized with a β-actin protein antibody (GTX-124214, 1:10,000; Genetex). Immuno-detection was carried out with an ECL kit (GE-Healthcare, Little Chalfont, UK) and the chemiluminescence signals were detected with ChemiDoc (Bio-Rad, Hercules, CA, USA).

### 4.8. In Silico Analysis of miRs-Target Interactions

The possible mRNA–miRNA interactions induced by ONC were obtained by comparing online databases of predicted interactions (DIANA-microT [92], ElMMo [93], MicroCosm [94], miRanda [95], miRDB [96], PicTar [97], PITA [98], TargetScan [99]), and databases of validated interactions (miRecords [100], miRTarBase [101], TarBase [102]) between selected miRNA and mRNA, using the Bioconductor package multiMiR [44], according to the reference manual.

To further enhance understandability, the overlapping of target genes of the mRNA–miRNA interactions were obtained using the R package VennDiagram [103].

### 4.9. miRs and Inhibitors Transfections

For inhibitors transfection, 150,000 cells were plated in 12-well plates. After 24 h, 1 µM ONC (final concentration) was added to the medium, and cells were transfected with miR-20a-3p (HSTUD0368) or miR-34a-5p (HSTUD0508) inhibitors (Merck, Darmstadt, Germany), or negative control (MIH00000) (ABM, Richmond, Canada) at 30 nM final concentration. Transfections were performed using lipofectamine 3000 (Thermo-Fischer Scientific), according to the manufacturer’s recommendations, and A375 cells were incubated for a further 72 h. 

For mimics transfection, 150,000 cells were plated in 12-well plates. After 24 h, miR-20a-3p (HM10368) or miR-34a-5p mimics (HM10508) (Merck, Darmstadt, Germany) or negative control (MCH00000) (ABM, Richmond, Canada), at 50 nM final concentration, were transfected using lipofectamine 3000 (Thermo-Fischer Scientific), according to the manufacturer’s recommendations, and incubated for 72 h.

### 4.10. Statistics

All the results are reported as a mean value ± SD. Unless otherwise noted, *p* values were determined using unpaired, two-tailed Student’s *t* test, with one asterisk, *, if *p* < 0.05, or two, **, if *p* < 0.01. For each type of experiment, a minimum of three independent biological replicates were performed.

## 5. Conclusions

We detected the ONC ability to upregulate the expression level of miR-20a-3p, miR-29a-3p, and miR-34a-5p, together with a downregulation of many miRNAs-associated oncoproteins. Notably, miRNAs stability depends on upstream regulation by long-non-coding-RNA or circular-RNA species that can be damaged by ONC ribonucleolytic activity. This could explain the pleiotropic anti-melanoma activity induced by ONC treatment.

Overall, these results shed light on the mechanisms underlying the anticancer activity in melanoma, and in tumor progression in general. 

## Data Availability

Not applicable.

## Supplementary Materials

The following supporting information can be downloaded at: https://www.mdpi.com/article/10.3390/ijms23031647/s1.
