# Supplementary material for: Upregulation of miR-34a-5p, miR-20a-3p and miR-29a-3p by Onconase in A375 Melanoma Cells Correlates with the Downregulation of Specific Onco-Proteins
Source: Int J Mol Sci. 2022 Jan 31;23(3):1647. doi: 10.3390/ijms23031647 (PMC8835754; doi:10.3390/ijms23031647)
Supplement: Supplementary file 1 [file ijms-23-01647-s001.zip › ijms-1527834-supplementary.pdf]

Supplementary data for

Upregulation of miR-34a-5p, miR-20a-3p and miR-29a-3p by Onconase in A375 melanoma cells correlates with the downregulation of specific onco-proteins

by

Elisa De Tomi [1,†], Rachele Campagnari [2,†], Elisa Orlandi [1,†], Alessia Cardile [2], Valentina Zanrè [2], ,Marta Menegazzi [2,*] , Macarena Gomez-Lira [1] and Giovanni Gotte [2]

[*]Corresponding Author

[†]Contributed equally

The data included are

Supplementary Table S1, Figure S1 and Figure S2

Table S1. miRs expression values recovered in the first experiment performed with ONC-treated or non-treated (control) A375 cells.

miRs	samples	Cq Mean	ΔCq	RQ
miR-30b-5p	+onconase	17.53	−0.30	1.23
miR-30b-5p	control	17.83		
miR-516b-5p	+onconase	30.33	−1.13	2.19
miR-516b-5p	control	31.46		
miR-146a-5p	+onconase	22.49	−0.83	1.77
miR-146a-5p	control	23.32		
miR-128-3p	+onconase	22.95	−1.40	2.64
miR-128-3p	control	24.35		
miR-34a-5p	+onconase	20.18	−1.54	2.91
miR-34a-5p	control	21.72		
miR-20a-3p	+onconase	21.23	−3.32	9.98
miR-20a-3p	control	24.54		
miR-20a-5p	+onconase	16.48	−1.64	3.12
miR-20a-5p	control	18.12		
miR-15b-5p	+onconase	17.60	−1.57	2.98
miR-15b-5p	control	19.18		
miR-24-3p	+onconase	17.09	−1.12	2.17
miR-24-3p	control	18.21		
miR-23a-5p	+onconase	N.A.	-	
miR-23a-5p	control	28.71		
miR-9	+onconase	22.93	−0.63	1.55
miR-9	control	23.56		
mir-29a-3p	+onconase	18.05	−1.80	3.48
mir-29a-3p	control	19.85		
miR-103a-3p	+onconase	23.28	−0.76	1.69
miR-103a-3p	control	24.04		
miR-27a-3p	+onconase	20.41	0.48	0.72
miR-27a-3p	control	19.93		
miR-106a-5p	+onconase	24.22	−1.17	2.25
miR-106a-5p	control	25.39		
miR-22-5p	+onconase	23.29	−2.33	5.03
miR-22-5p	control	25.62		
miR-941	+onconase	25.44	1.13	0.80
miR-941	control	24.31		
miR-21-5p	+onconase	15.42	−2.08	4.24
miR-21-5p	control	17.50		
miR-532-5p	+onconase	22.88	1.45	0.37
miR-532-5p	control	21.43		
miR-140-5p	+onconase	22.36	−0.40	1.32
miR-140-5p	control	22.76		
miR-326	+onconase	28.17	−0.14	1.10
miR-326	control	28.31		
miR-425-5p	+onconase	21.37	0.06	0.96
miR-425-5p	control	21.31		
miR-495-3p	+onconase	30.12	−0.25	1.19
miR-495-3p	control	30.36		
miR-191-5p	+onconase	19.25	0.23	0.85
miR-191-5p	control	19.02		

N.A. = not amplified; RQ = relative quantity.

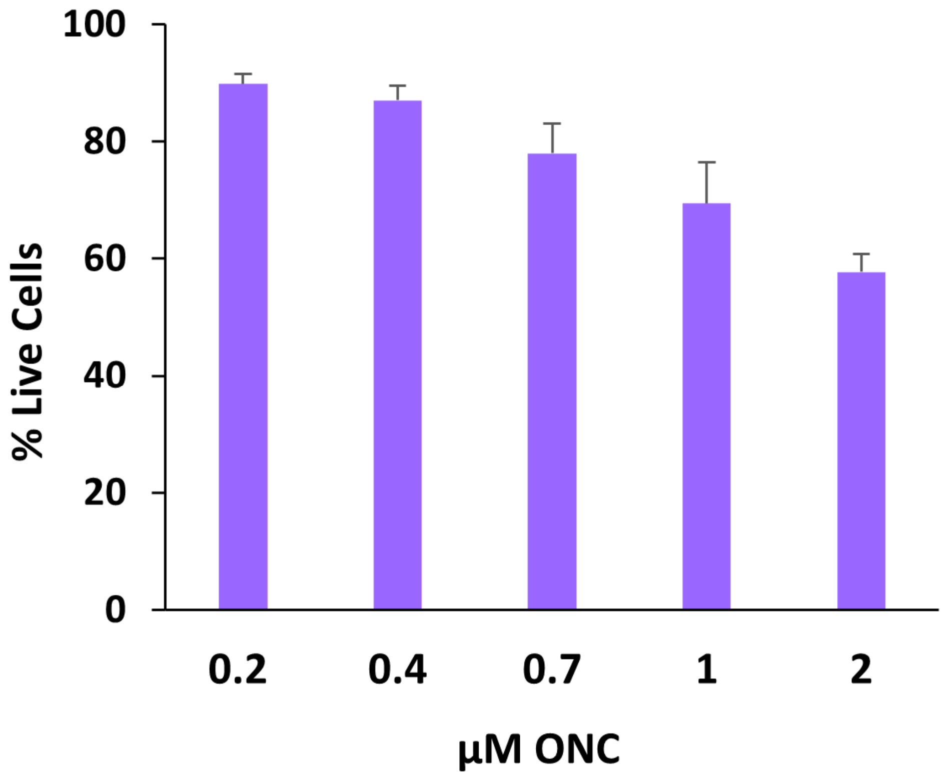

Figure S1. Cell viability assay of FO1 cells treated with different ONC concentrations. Cells were seeded in 96-well plates (2.9×10^3 cells/well). After 24 h, cells were incubated for 72 h with different concentrations of ONC. At the end of the treatment, cells were fixed by adding 25 µL/well of 50% (W/v) TCA directly into the culture medium. Plates were incubated at 4 °C for 1 h, washed four times with ddH_2O and dried at RT. Staining was performed by adding 50 µL/well of 0,04% (W/v) sulforhodamine B (SRB) sodium salt solution (Sigma-Aldrich, Milan, Italy). After 1 h RT incubation, plates were rinsed with 1% HAc and air-dried. SRB was solubilized in 10 mM Tris-base solution pH 10.5 and Abs_{510} measured in the plate reader TECAN NanoQuant Infinite M200 Pro (Tecan Group Ltd.). Four replicates for each condition/data point were performed.

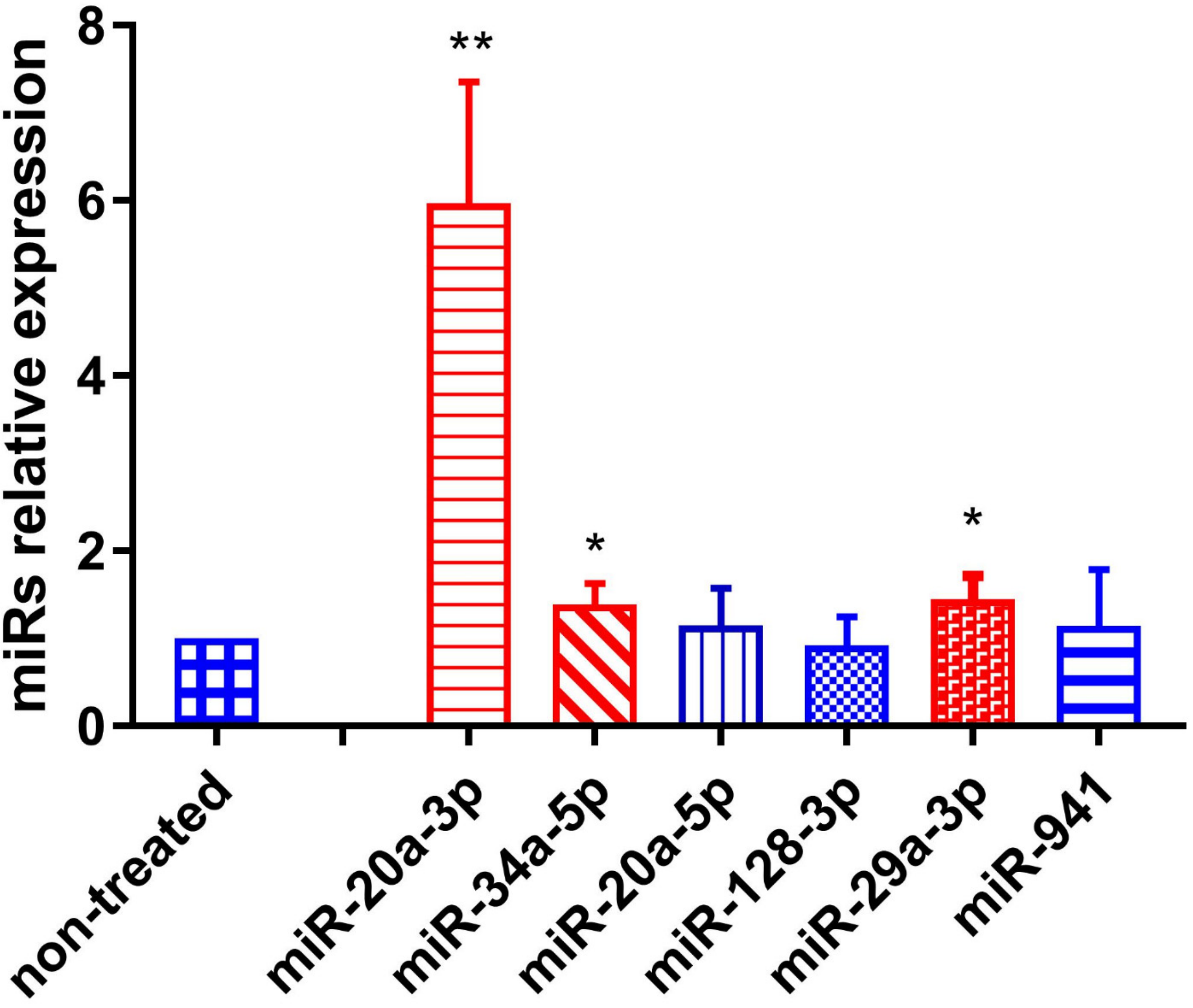

Figure S2. Relative expression of miRNAs after a 48 h ONC incubation of FO1 cells. FO1 cells were cultured for 48 hours after 1 μM ONC administration in the culture medium. Red color bars refer to the onco-suppressor miRNAs that are upregulated by ONC at a statistically significant level; the blue ones refer instead to miRNAs whose expression level after ONC is not significantly different from untreated control. The mean values ± S.D. of miRs expression level measured by RT-PCR and deriving from three independent experiments are shown. All comparisons were performed *vs* each control sample after normalization to miR-191 expression; * $p < 0.05$, ** $p < 0.01$.
